# Trends and Seasonal Patterns of Intussusception in Japan: A Retrospective Database Study of Weather and Infectious Disease Associations

**DOI:** 10.7759/cureus.91808

**Published:** 2025-09-07

**Authors:** Yoshiaki Kabata, Hiroyuki Namba

**Affiliations:** 1 Ophthalmology, Jikei University School of Medicine, Daisan Hospital, Tokyo, JPN; 2 Pediatrics, Jikei University School of Medicine, Kashiwa Hospital, Chiba, JPN

**Keywords:** covid-19, intussusception, japan, pharyngoconjunctival fever, seasonality, weather

## Abstract

Background

Intussusception is a primary cause of acute abdomen in young children, often requiring surgical intervention. However, its etiology is largely unknown. This study aimed to analyze trends and seasonality of intussusception procedures in Japan using a national database and to investigate their association with weather conditions and pediatric infectious diseases.

Methods

This retrospective study utilized the National Database of Health Insurance Claims and Specific Health Checkups of Japan (NDB) to analyze data on intussusception reduction procedures from fiscal years 2014 to 2022. Monthly meteorological data and pediatric infectious disease reports were collected for the same period. Statistical analyses, including the Wilcoxon signed-rank test, Pearson correlation, and partial least squares (PLS) regression, were performed on data from 2020 to 2022.

Results

The annual number of intussusception reduction procedures decreased from 5,564 in 2014 to 2,342 in 2022, with a sharp decline to 2,056 cases in 2020. A significant seasonal variation was observed, with a higher incidence in summer compared to spring (p=0.0134) and winter (p=0.0243). Significant positive associations were found between the number of intussusception reduction procedures and temperature (r = 0.5102, p = 0.0015; PLS coefficient = 0.3663; variable importance in projection (VIP) = 1.2466), relative humidity (r = 0.5479, p = 0.0005; PLS coefficient = 0.2479; VIP = 1.273), and pharyngoconjunctival fever (r = 0.5723, p = 0.0003; PLS coefficient = 0.5273; VIP = 1.6386).

Conclusions

This study revealed a declining trend in intussusception reduction procedures in Japan, with a notable decrease in 2020 potentially linked to the COVID-19 pandemic. The incidence peaked in the summer, showing strong associations with higher temperature, relative humidity, and pharyngoconjunctival fever outbreaks. These findings can contribute to developing more effective prevention and early diagnosis strategies for intussusception.

## Introduction

Intussusception is a leading cause of acute abdomen in children, especially infants, and a serious condition that can lead to intestinal necrosis and death if left untreated [1-4]. While surgical intervention, including resection, is often required, the etiology of intussusception remains largely unknown, and it is often classified as idiopathic [1, 2]. Previous studies have suggested that there may be seasonal patterns in the occurrence of intussusception. For example, some studies from China [5-7] and India [8] have reported a trend of increased cases during the summer months, while others, such as those from Israel [3] and Pakistan [9], have found no seasonality. This suggests that the seasonality of intussusception may vary depending on region and climatic conditions [2, 5].

Factors like temperature, humidity, and other weather conditions, as well as viral infections, have been suggested as potential contributing factors [6]. In particular, certain infections, such as adenovirus, are suspected of increasing the risk of intussusception [10, 11]. While understanding regional trends is important, comprehensive data for Japan is currently lacking [12].

The Ministry of Health, Labor and Welfare (MHLW) of Japan operates the National Database of Health Insurance Claims and Specific Health Checkups of Japan (NDB), which contains most of the data on the national healthcare insurance system [13]. As Japan has a universal health insurance system, the NDB data is comprehensive and representative of medical care trends nationwide [13]. The purpose of this study was to examine the number of intussusception procedures using the NDB database and to analyze annual and seasonal changes. Furthermore, we aimed to investigate the relationship between the number of intussusception procedures and weather conditions and pediatric infectious diseases in Japan. Understanding these interactions may contribute to the development of more effective prevention and early diagnosis strategies for intussusception.

## Materials and methods

This retrospective, descriptive study used the NDB database [13]. All investigations adhered to the principles of the Declaration of Helsinki.

The NDB database contains a single code for the intussusception procedure: K751 (intussusception reduction), which was used for data extraction in this study. The number of intussusception reduction procedures was collected from the NDB database for fiscal years 2014 to 2022. The fiscal year and month correspond to the calendar year and month. Monthly NDB data were obtained from fiscal years 2019 to 2022, as monthly data became available in 2019. Data on live births from 2014 to 2022 were obtained from the Ministry of Internal Affairs and Communications [14].

Monthly meteorological data, including temperature (°C), precipitation (mm), all-day solar radiation (MJ/m²), wind speed (m/s), relative humidity, and atmospheric pressure (hPa), were collected from the Japan Meteorological Agency database [15]. Data from Tokyo, Nagoya, Osaka, and Fukuoka were averaged, as these regions account for a large proportion of intussusception reduction procedures.

Data on the monthly number of pediatric infectious disease reports were obtained from the Surveillance of Infectious Disease Outbreak Trends database managed by the National Institute of Infectious Diseases [16]. The following diseases were included: influenza, respiratory syncytial virus infection, pharyngoconjunctival fever, group A streptococcal pharyngitis, infectious gastroenteritis, chickenpox, hand, foot, and mouth disease, erythema infectiosum, exanthem subitum, herpangina, and rotavirus. These data are based on fixed-point observations, not all-counts surveys [16].

Seasons were defined as spring (March-May), summer (June-August), fall (September-November), and winter (December-February) [17]. The statistical examination of monthly intussusception reductions was performed from 2020 to 2022. The year 2019 was excluded from this specific analysis due to a significant drop in cases from 3,948 in 2019 to 2,056 in 2020.

To analyze seasonal differences, the Wilcoxon signed-rank test was used. Pearson correlation and partial least squares (PLS) regression were performed to analyze the relationship between the monthly number of intussusception reduction procedures, weather conditions, and pediatric infections. Statistical significance was set at p < 0.05. In the PLS regression model, variable importance in projection (VIP) scores greater than 1.0 were considered influential. All statistical analyses were performed using JMP® Pro 16 (SAS Institute Inc., Cary, NC).

## Results

**Figure 1:**
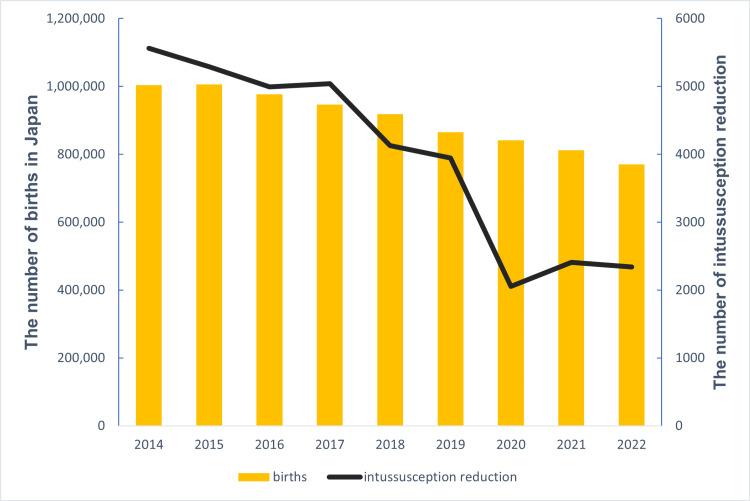
Trends in the number of births and intussusception reduction procedures in Japan from 2014 to 2022 The annual number of births is represented by the bar graph (left y-axis), while the annual number of intussusception reduction procedures is shown by the line graph (right y-axis). Birth data were obtained from the Ministry of Internal Affairs and Communications, and intussusception data were sourced from the National Database of Health Insurance Claims and Specific Health Checkups of Japan (NDB).

Figure 1 shows the trends in the number of live births and intussusception reduction procedures in Japan from 2014 to 2022. The number of intussusception reduction procedures decreased by more than half, from 5,564 in 2014 to 2,342 in 2022. The lowest number of cases was recorded in 2020, with 2,056 cases. This decline, particularly the marked decrease in 2020, was more significant than the concurrent decline in the birth rate.

**Figure 2:**
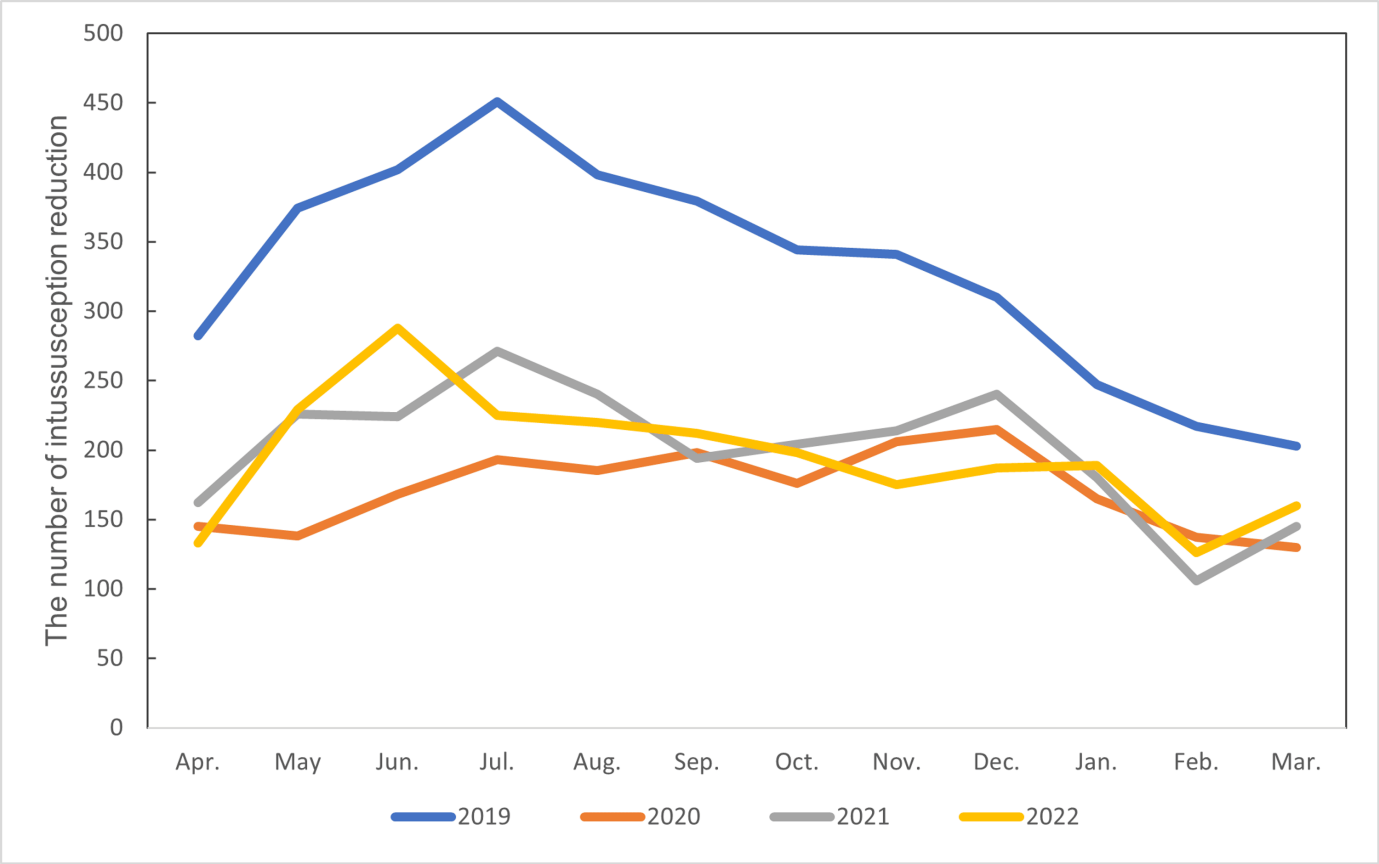
The monthly number of intussusception reduction procedures from 2019 to 2022 A notable decrease in cases is evident from 2020 onwards compared to the pre-pandemic level in 2019.

Figure 2 illustrates the monthly number of intussusception reduction procedures from 2019 to 2022. Consistent with the annual trend, a sharp decrease in the number of cases was observed starting in 2020.

**Figure 3:**
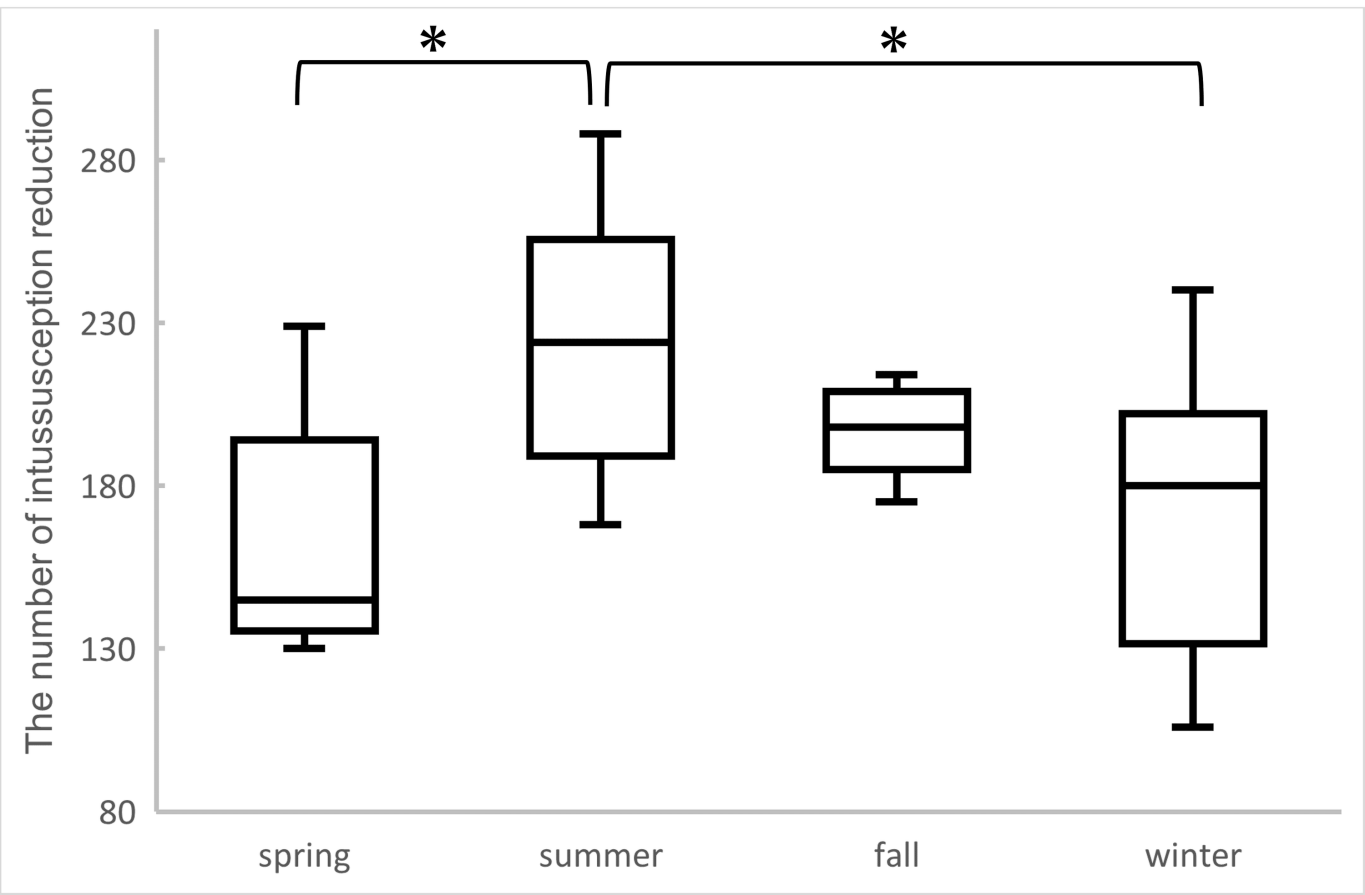
Box-and-whisker plot of the number of intussusception reduction procedures by season from 2020 to 2022 The seasons were divided into spring (March, April, and May), summer (June, July, and August), fall (September, October, and November), and winter (December, January, and February). Significant differences were observed between summer and spring (p=0.0134) and summer and winter (p=0.0243) using the Wilcoxon signed-rank test. No significant differences were found among the other seasons.

Figure 3 presents a box-and-whisker plot of the number of intussusception reduction procedures by season from 2020 to 2022. The Wilcoxon signed-rank test revealed significant differences between summer and spring (p=0.0134) and between summer and winter (p=0.0243). No significant differences were found among the other seasons.

**Figure 4:**
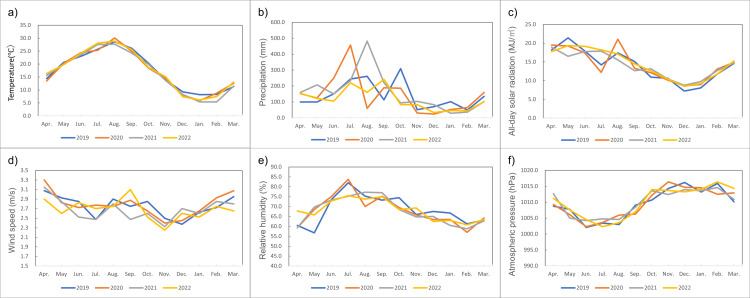
Monthly weather conditions in Japan from 2019 to 2022 a): temperature (°C); b): precipitation (mm); c): all-day solar radiation (MJ/㎡); d): wind speed (m/s); e): relative humidity; f): atmospheric pressure (hPa). Weather conditions’ data from Tokyo, Nagoya, Osaka, and Fukuoka were used as an average [15].

Figure 4 provides line graphs of monthly weather conditions in Japan from 2019 to 2022.

**Figure 5:**
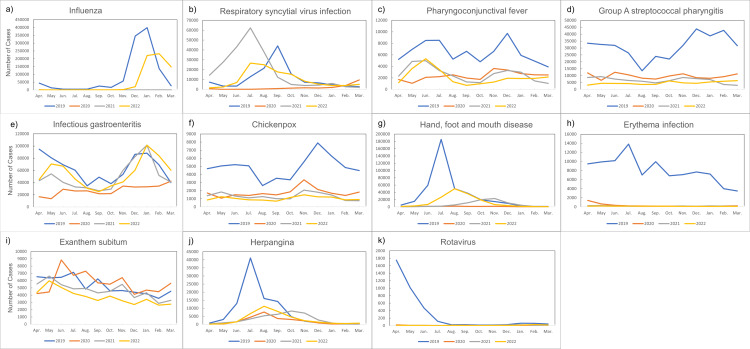
The number of monthly pediatric infectious diseases in Japan from 2019 to 2022 a): influenza; b): respiratory syncytial virus infection; c): pharyngoconjunctival fever; d): group A streptococcal pharyngitis; e): infectious gastroenteritis; f): chickenpox; g): hand, foot, and mouth disease; h): erythema infection; i): exanthem subitem; j): herpangina; and k): rotavirus Pediatric infectious diseases’ data were obtained from the Surveillance of Infectious Disease Outbreak Trends. These are not all-counts surveys but fixed-point observations [16].

Figure 5 provides line graphs of the number of monthly pediatric infectious diseases in Japan from 2019 to 2022.

Table 1 displays the results of the Pearson correlation and PLS regression analyses examining the relationship between the number of intussusception reduction procedures, weather conditions, and pediatric infections. The PLS model was constructed using non-linear iterative partial least squares (NIPALS), with seven components selected based on the percentage of variance explained and the root mean squared error of prediction, which explained 83.2% of the variance in seven components. The predicted residual error sum of squares (PRESS) mean square root had a minimum value of 0.83338; the root mean squared error of prediction was 0.73. Significant positive associations were found between the number of intussusception reduction procedures and temperature (r = 0.5102, p = 0.0015; PLS coefficient = 0.3663; VIP = 1.2466), relative humidity (r = 0.5479, p = 0.0005; PLS coefficient = 0.2479; VIP = 1.273), and pharyngoconjunctival fever (r = 0.5723, p = 0.0003; PLS coefficient = 0.5273; VIP = 1.6386).

**Table 1:** Pearson correlation coefficient and partial least squares (PLS) regression to analyze the relationship between the number of intussusception reduction procedures, weather conditions, and pediatric infections per month Statistical significance was set at P < 0.05. Variable importance in projection scores (VIP) greater than 1.0 was considered influential. There was a significant association between the number of intussusception reduction procedures and temperature (r = 0.5102, p = 0.0015, PLS coefficient = 0.3663, VIP = 1.2466), relative humidity (r = 0.5479, p = 0.0005, PLS coefficient = 0.2479, VIP = 1.273), and pharyngoconjunctival fever (r = 0.5723, p = 0.0003, PLS coefficient = 0.5273, VIP = 1.6386).

Variables	Pearson correlation		PLS regression	
	r	p	PLS coefficient	VIP
Temperature (°C)	0.5102	0.0015	0.3663	1.2466
Precipitation (mm)	0.2778	0.1009	-0.0311	0.8497
All-day solar radiation (MJ/㎡)	0.0854	0.6204	-0.1573	0.6579
Wind speed (m/s)	-0.3557	0.0332	-0.0383	1.0481
Relative humidity (%)	0.5479	0.0005	0.2479	1.273
Atmospheric pressure (hPa)	-0.4546	0.0053	0.0387	1.1117
Influenza	-0.2289	0.1794	-0.0098	0.6148
Respiratory syncytial virus infection	0.5356	0.0008	0.0872	1.2341
Pharyngoconjunctival fever	0.5723	0.0003	0.5273	1.6386
Group A streptococcal pharyngitis	-0.115	0.5042	-0.1593	0.4181
Infectious gastroenteritis	0.0851	0.6216	0.3209	0.9845
Chickenpox	0.1213	0.4808	0.2083	0.6297
Hand, foot, and mouth disease	0.3529	0.0348	0.0552	0.8749
Erythema infection	-0.2655	0.1175	0.2166	0.9323
Exanthem subitum	0.2104	0.218	-0.1833	0.7058
Herpangina	0.4434	0.0068	0.2098	1.0325
Rotavirus	-0.4156	0.0117	-0.2391	1.0053

## Discussion

This study demonstrates a declining trend in the number of intussusception reduction procedures in Japan, with a particularly sharp decrease in 2020. This trend may be influenced by several factors, including the declining birth rate and improvements in pediatric healthcare. However, the significant drop in 2020 strongly suggests an impact from the COVID-19 pandemic. While patients with an emergency condition like intussusception would likely not avoid seeking medical care [18], preventive measures for COVID-19 may have reduced the incidence of preceding viral infections associated with intussusception. Similar decreases in intussusception cases in 2020 have been reported in South Korea [19], the United States [20], Australia [21], and Tokyo [22], supporting this hypothesis.

Our analysis of monthly data from 2020 to 2022 revealed a significant peak in incidence during the summer compared to spring and winter. This finding is consistent with reports from other Asian regions like India [8] and Taiwan [23], although studies in other areas have found no seasonality [3, 9], highlighting potential regional differences. We identified temperature, humidity, and pharyngoconjunctival fever as influential factors for this seasonality. The association with climatic factors aligns with previous studies showing positive correlations between intussusception and temperature, humidity, and other meteorological parameters [5, 8, 23].

While the etiology of intussusception remains unclear, infectious pathogens, particularly adenovirus, are widely implicated [1, 10, 11, 24]. Adenovirus is thought to cause hyperplasia of lymphoid tissue in the intestinal wall, leading to intussusception [11, 25-28]. Our study found a strong association between intussusception and pharyngoconjunctival fever, a syndrome primarily caused by adenovirus. This finding indirectly supports the link between adenovirus and intussusception, consistent with previous reports [11, 26]. By examining the association with a specific infectious disease rather than just the pathogen, we can potentially gain insights into the interplay of various pathogens and leverage publicly available disease outbreak data for timely public health alerts.

This study has several limitations. First, the NDB database only provides the number of procedures and lacks detailed clinical information for each case. Second, the meteorological data were monthly averages from four major cities, which may not capture localized or rapid weather changes. Third, the infectious disease data were nationwide totals, potentially masking regional variations. Finally, this study demonstrates correlation, not causation. Further research with more granular, localized data is needed to confirm these findings and explore the impact of factors like the rotavirus vaccine [29], which was not assessed in this study.

## Conclusions

In conclusion, this study demonstrated that the number of intussusception reduction procedures in Japan has generally declined over recent years, with an especially marked decrease observed in 2020 that coincided with the COVID-19 pandemic. Beyond this overall trend, we identified a clear seasonal variation, with a pronounced peak during the summer months that was closely associated with higher temperature, increased relative humidity, and concurrent outbreaks of pharyngoconjunctival fever. Taken together, these results provide new insights into both the temporal and environmental factors influencing intussusception occurrence in Japan. A deeper understanding of these patterns may contribute to the development of effective prevention strategies, guide clinicians in anticipating seasonal surges, and ultimately support more timely diagnosis and management for affected children.
